# An emerging role for tissue plasticity in developmental precision

**DOI:** 10.1042/BST20230173

**Published:** 2024-05-08

**Authors:** Sundar Ram Naganathan

**Affiliations:** Department of Biological Sciences, Tata Institute of Fundamental Research, 1, Dr. Homi Bhabha Road, Colaba, Mumbai 400005, India

**Keywords:** feedback, mechanics, precision, robustness, somite, symmetry

## Abstract

Reproducible tissue morphology is a fundamental feature of embryonic development. To ensure such robustness during tissue morphogenesis, inherent noise in biological processes must be buffered. While redundant genes, parallel signaling pathways and intricate network topologies are known to reduce noise, over the last few years, mechanical properties of tissues have been shown to play a vital role. Here, taking the example of somite shape changes, I will discuss how tissues are highly plastic in their ability to change shapes leading to increased precision and reproducibility.

## Introduction

A prevalent feature of embryonic development is the robustness of tissue morphogenesis, where reproducible morphology emerges in a tissue from the same species. Robustness emerges despite the presence of noise emerging from the inherent stochasticity in signaling, molecular interactions and gene expression, in addition, to genetic and environmental variation in a population. To understand the mechanisms conferring robustness in a system, precise quantitative measurements of the entity being probed needs to be undertaken after careful consideration of the different sources of noise and variation [1]. Robustness in general is said to be conferred by several mechanisms including the expression of genes with redundant functions, through multiple signaling pathways together contributing towards a common functional output, also known as distributed robustness [2], gene organization in the chromosome [3], as well as through intricate network topologies including gene regulatory networks [4] that comprises feedback and feed-forward loops [5, 6]. Over the last decade, tissue mechanics has added a new chapter in the context of emergence of precise and reproducible tissue morphologies [7–13].

In this mini-review, I will discuss the role of tissue mechanics in developmental precision with a particular focus on the emergence of left–right symmetry in somites followed by directing attention to a few examples where mechanics has been demonstrated to buffer intrinsic noise in the context of shape and size regulation. Left–right symmetry, also known as bilateral symmetry, is a prevalent feature across most animals where similar tissue shapes and sizes form within the same individual, for example in paired tissues such as somites, ears and eyes that develop on the left and right sides of the embryo. Studying how precision emerges on the two sides in the same individual is beneficial as genetic and environmental variation can be safely ignored likely enabling the discovery of processes that buffer unavoidable intrinsic noise in the system.

## Tissue mechanics drives increased precision in somite lengths

Somites, the precursors of most of the adult muscle and skeletal system, are left–right symmetric tissues that emerge during embryonic development. Somite morphogenesis has been extensively studied across species and the underlying mechanisms driving somite formation in a rhythmic and sequential manner are being increasingly parsed out in detail. Briefly, somite formation is driven by a segmentation clock that comprises gene transcriptional oscillations of the Hes/Her gene family resulting from negative feedback loops [14–16] and synchronization of this transcriptional state among a local group of cells via Delta–Notch signaling that depends on a combination of positive and negative feedback loops [17]. At the tissue-scale, this is manifested as a wave of transcription that traverses from the posterior of the embryo to the presumptive position where the next somite pair forms in the anterior. The exact mechanism that converts the temporal information carried by the waves to a spatial information that sets the initial size of a somite are, however, still hotly debated, with roles for an intrinsic timer that regulates the clock [18], Wnt [19] and retinoic acid morphogen gradients [20, 21] as well as clock-mediated periodic inhibition of FGF signaling [22] being proposed.

Somites are three-dimensional units of groups of epithelial and mesenchymal cells [23]. The size of a somite, largely described only in the form of its length along the anteroposterior (AP) axis (also known as AP length), has a stereotypical pattern along the body axis. In all species examined, the initial AP length in the anterior somites are small, the trunk somites are relatively bigger, followed by a gradual decrease in length towards the posterior somites [24, 25]. Many of the above-mentioned processes that drive somite formation have an effect on the initial AP length. A slowdown [26] or increasing the speed of the clock [27] leads to a corresponding increase or decrease in AP length, respectively, and similarly, inhibition or activation of the various signaling gradients [19, 28, 29] and synchronization processes [30] results in a change in AP length. Although not studied in detail, the precision with which the initial AP length of a somite is set is, therefore, likely to depend on the network topology and the strength of interactions between these different processes.

**Figure 1.**
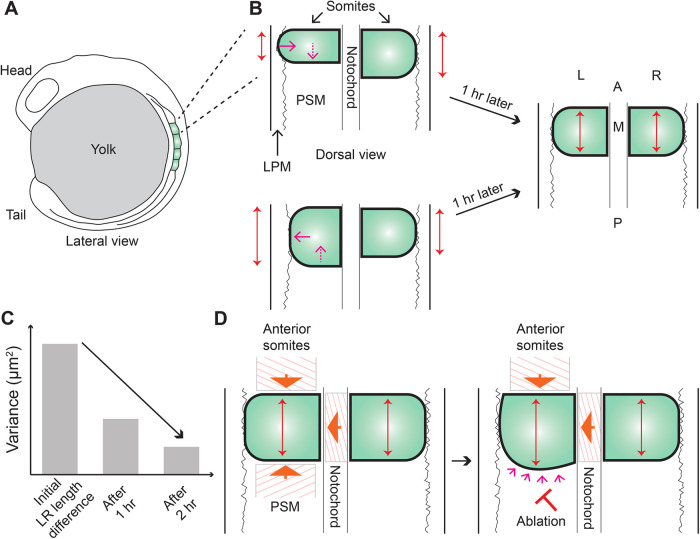
Somite AP lengths get precise over time. (**A**) Representation of a zebrafish embryo at the four-somite stage. Somites are marked in green. (**B**) Somites often form with imprecise anteroposterior (AP) lengths (compare length of red double-headed arrows between the left and right somites), which get corrected over time (right panel). A change in AP length is buffered by a corresponding change in mediolateral (ML) length. The lateral boundary of somites is relatively curved compared with other boundaries. PSM, presomitic mesoderm; LPM, lateral plate mesoderm; M, medial; L, left; R, right; A, anterior; P, posterior; red arrow, direction of ML length change; dashed arrow, direction of AP length change. The cartoons are representative of anterior somites in zebrafish embryos. (**C**) The initial differences in length between the left and right somite pairs are highly variable, which is a readout of inherent noise in somite formation. The variability in the length difference decreases over time (indicated by the arrow). (**D**) External contact stresses from different directions (indicated by arrows) play a role in determining the shape of a somite. If the stress is relieved by laser ablation (for example in the PSM as indicated in the right panel), the boundary adjacent to the ablated region bulges over time (small arrows indicate bulging).

Importantly, even in an unperturbed system, i.e. in wild type, there still exists substantial variability in the initial AP length of somites as quantified recently in zebrafish embryos [8] (Figure 1A–C). This intrinsic variability can be considered to be a measure of the collective noise in the upstream signaling and transcriptional processes that drive somite formation. Zebrafish has 32 somites along the body axis and the variability in somites 1–8 were analyzed in this study and discussed in detail below. The initial AP length of many somites varied by 15–20% compared with the mean across embryos and this variability was observed even for the same somite ordinal number across the left and right sides indicating the presence of initial asymmetries. Although not explicitly mentioned, a similar extent of variability has been previously observed in a few studies across species [19, 24, 25, 29]. It has been previously reported that a consistent 6–7% increase in somite length along the body axis can lead to lasting effects in the musculoskeletal system with a reduced number of skeletal elements [26]. A much larger left–right difference in somite lengths observed in wild-type embryos [8] could, therefore, potentially lead to left–right asymmetries in adults, but such asymmetries persisting from the embryonic stage into adulthood have never been reported in wild type.

How do embryos then buffer such variability to ensure minimal long-lasting effects? Interestingly, by following the same somites through development, it was observed that the variability in somite length decreased over time, with AP lengths getting adjusted towards a precise target length even within the same embryo [8]. As a consequence of this adjustment, AP lengths on the two sides become similar over time, thus ensuring increased left–right symmetry (Figure 1B,C). This observation of precision in tissue size emerging over time is not exclusive to somites and has been previously reported in left–right otic vesicle (precursors of the ear) development in zebrafish, where a decrease in variability of tissue size from 16 hours post fertilization (hpf) to 48 hpf was shown to result in increased symmetry in otic vesicle morphology [31]. Notably, in both cases, the correction mechanism was shown to be independent of processes that drive upstream morphogenesis of the respective tissues. In the case of otic vesicle, perturbation of FGF signaling, an important regulator of initial ear size, led to increased asymmetries but still the embryos recovered to attain symmetry by 48 hpf. Similarly, in somites, despite mild perturbations to the clock and the synchronization process, which led to a different initial size and increased variability, respectively, length adjustments still proceeded normally [8]. Furthermore, adjustment continued unperturbed on one side even in the total absence of contralateral somites suggesting that somites attain a target length independently on the two sides [8], a phenomenon that is also well appreciated in left–right otic vesicle [31, 32] and limb bud development [33]. In early embryos, it, therefore, appears that minimal cross-talk is required between the two sides to result in tissues of precise sizes.

What drives the size control of a somite on each side? During the time period of AP length adjustment, the volume of a somite remained unchanged, which directly pointed to the role of shape changes in a somite [8]. Accordingly, it was observed that a change in AP length was highly correlated to a corresponding change in mediolateral (ML) length (Figure 1B). To test whether neighboring tissues play a role in this shape transition or if this is entirely intrinsic to a somite, ablation experiments were performed. Ablating the presomitic mesoderm immediately posterior to the most-recently-formed somite resulted in a bulging of the nearest somite boundary (Figure 1D). This suggested the presence of significant contact stresses along the AP axis, which when relieved by ablation resulted in somite shape changes (Figure 1D). A similar observation was made in chick embryos, where somites bulged into regions devoid of neighboring somites that were mechanically removed [34]. In contrast, ablating the lateral plate mesoderm, a tissue that is present lateral to somites, did not result in any somite shape changes, suggesting relatively lesser constraint on somites laterally. A consequence of this could be readily observed in the shape of the lateral somite boundary, which tends to be curved, in contrast with the relatively straighter somite boundaries abutting other tissues (Figure 1). In addition, this provides a possible explanation for the ML length majorly buffering adjustments in AP length, as this dimension is relatively free to change shape given the lesser constraint, particularly in anterior somites. Whether the overlying ectoderm, which abuts the lateral plate mesoderm further away from the somites, also plays a role is yet to be tested.

Although not mapped out in detail, as the shape of a somite changed, concomitantly, cells within anterior somites were observed to undergo rearrangements akin to a diffusive process [8]. It is important to note here that shape changes in anterior somites immediately after formation were probed in this study when minimal cell shape changes are observed. The mechanism by which precision emerges could be different, however, in posterior somites, where upon somite formation, cells within a somite in addition undergo substantial cell shape changes [35]. Together, these observations indicate that somites attain a precise AP length that stems from a cross-talk, which remains to be characterized in detail, between external contact stresses from neighboring tissues and internal active forces that drive cell rearrangements. Given a set size for the entire presomitic mesoderm, it was also proposed that adjustments of length will automatically lead to positional symmetry of somites [8]. However, what ensures a similar size of the presomitic mesoderm on the two sides is unknown, although at later somite stages, the symmetric flow of cells into the presomitic mesoderm could play a role [36].

## Tissue plasticity is vital for developmental precision

To understand shape changes in a tissue quantitatively, it is imperative to be cognizant on the material property of a tissue. Of late, it appears that cells and tissues can be considered as yield stress materials [37–41]. The yield stress of a tissue corresponds to the maximum mechanical stress it can withstand in a solid-like state, before cells within the tissue start to rearrange and flow thereby permanently changing the shape of a tissue. This regime characterized by a permanent change in the shape of a material (in this context, read as ‘tissue’) even when the stress is relieved represents the plasticity of a material [42].

**Figure 2.**
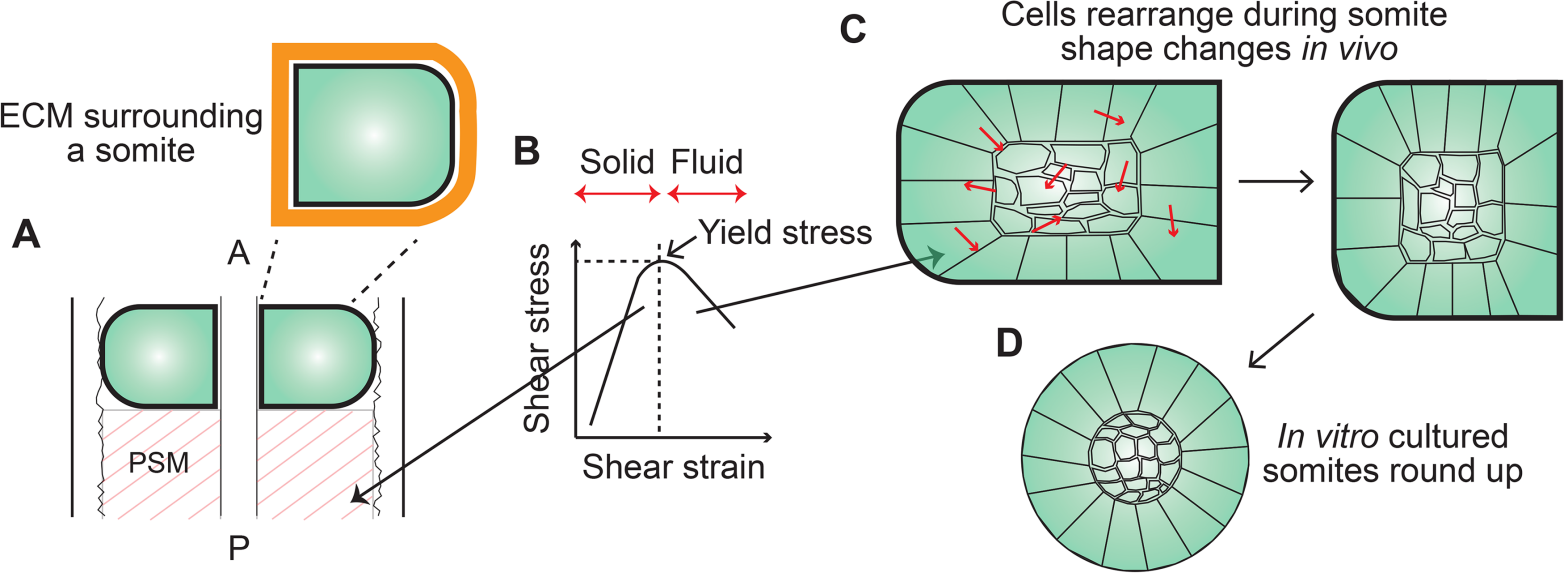
Somite tissue is in a fluid-like state. (**A**) Schematic of an anterior somite pair with the presomitic mesoderm (PSM) marked. Zoomed in image (top right) shows the presence of extracellular matrix (ECM, orange) around a somite. (**B**) Plot of shear stress and shear strain for a yield stress material. Below the yield stress value (marked in the plot), a material exhibits a solid-like state, while above the yield stress, fluid-like behavior is observed. The anterior PSM is in a solid-like state (long black arrow towards the left), while somites exhibit fluid-like behavior (long black arrow towards the right). (**C**) As a somite changes shape *in vivo* (for simplicity surrounding tissues are not shown), cells within the somite rearrange (red arrows) indicating a fluid-like state. The boundary cells are epithelial in nature, while the inner core consists of mesenchymal cells and this depiction is representative of anterior somites. (**D**) A somite isolated from a zebrafish embryo and cultured *in vitro*, which is devoid of neighboring tissues, tends to round up, indicating the presence of significant surface tension.

What makes a tissue plastic? In the context of somites, as mentioned above, during the time period of somite shape change, cells within a somite were observed to rearrange with a diffusive dynamics over the time-scale of length adjustment [8] (Figure 2C). One can, therefore, argue that tissues have to be in a fluid-like state to exhibit plasticity, i.e. lasting shape changes. In contrast, recent experiments by injection of ferrofluid droplets in zebrafish embryos to directly measure cellular and supracellular stresses in the anterior presomitic mesoderm just before somites form, have shown the tissue to behave as a yield stress material with a solid-like state [37] (Figure 2B). One possible explanation for the observed difference in [8] is that once a somite forms and an extracellular matrix encapsulates it (Figure 2A), sufficient stresses are generated to drive the somite tissue beyond its yield stress and, therefore, towards a fluid-like state. Accordingly, an isolated somite was observed to round up within 30 min of explanting the tissue (Figure 2D), signifying the role of surface tension in generating sufficient stresses to fluidize the tissue [8]. Furthermore, perturbations to molecules implicated in surface tension slowed down the observed shape changes validating this claim. However, direct measurements of somite surface tension are yet to be performed, which will be crucial to resolve the debate on the material state of the somite tissue leading to the observed shape changes. The detailed molecular activities that drive cell and tissue shape changes as well as a general overview of tissue mechanics will, however, not be discussed here and I will instead refer to extensive reviews previously written on these topics [43–53].

In general, tissues can also change shape without accompanying cell rearrangements, where changes in tissue shape can be directly correlated with concomitant changes in cell shapes. For example, during *Drosophila* ventral furrow formation, local transient cell shape changes, known as apical constriction, driven by the apical actomyosin cortex, lead to an out-of-plane bending of the tissue [54]. Notably, even though no cell rearrangements have been reported in this system, the resulting tissue deformation seems to be dependent on a viscous response of the cytoplasm to the force generated apically [55]. Thus, irrespective of the precise material property of the tissue, a fluid-like state of the tissue seems to be important for mediating shape changes. Other examples where a fluid-like state of tissues have shown to be important for tissue shape changes include the spreading of the zebrafish blastoderm before gastrulation [56, 57], epithelial gap closure during insect gastrulation [58], tissue flows during avian gastrulation [59], elongation of the zebrafish tail bud [36, 60], convergence–extension during germ band elongation in *Drosophila* [61] and Kupffer’s vesicle morphogenesis in zebrafish [62]. Tissue fluidization in fact is important for various other contexts of morphogenesis such as the rotational motion of connected cells in the *Drosophila* eye [63], wound healing [64] and collective cell migration [65–67]. Most of the above-mentioned shape transitions emerge from regulated changes in gene expression patterns that in turn drive shape changes in tissues. For example, the initial shape changes important for apical constriction are driven by spatiotemporally regulated expression of specific transcription factors [54]. Similarly, zebrafish somites also change from a coarsely cuboidal to a chevron shape [35, 68] over time, which is regulated by a coordination of FGF and Shh signaling pathways [69]. In contrast, the subtle shape changes that were observed in [8] purely emerge as a response to a particular mechanical environment given an initial state and can be considered to be a case of self-organization.

Notably, as mentioned in the previous section, changes in somite shape are important to ensure increased precision and symmetry. The prevailing idea here is that the upstream segmentation clock-related processes set a crude initial AP length, which gets fine-tuned by tissue mechanics to result in increased precision. In other words, tissue mechanics serves to buffer collective noise emerging from transcriptional and signaling processes, an emerging theme across diverse contexts. For example, the cephalic furrow, an invagination that forms all along the dorsal–ventral axis in gastrulating *Drosophila* embryos, is known to form in a straight manner without the appearance of any kinks or branching. This precise formation of the furrow is dependent on tissue-scale mechanical coupling [70] that buffers spatiotemporal heterogeneities in furrow initiation as well as the inherent noise in generating contractile forces [9]. Similarly, mechanical forces ensure the straightness of other morphological boundaries such as the dorsoventral compartment boundary in the *Drosophila* wing disc [71], parasegmental boundaries in early *Drosophila* embryos [72] and the notochord-presomitic mesoderm boundary in zebrafish embryos [73].

Tissue mechanics is also important for maintaining precise shapes in the presence of heterogeneities in a developing organ. For example, sepal growth in *Arabidopsis* flowers is driven by cell proliferation and if the position of the dividing cells and the rate at which they divide are spatially heterogeneous, which do occur, sepal shapes can get deformed over time. However, during the growth phase, sepals are known to largely maintain their shape and this was shown to depend on spatiotemporal averaging of cellular growth [74] as well as on a stress-feedback mechanism, where dividing cells are mechanically shielded by a ring of microtubules spread across the immediate neighbors [10], ensuring robust maintenance of shapes.

Finally, tissue mechanics is important for robust contralateral cell and pattern matching during development. This has been demonstrated in *Drosophila* embryos in two different contexts. One, during dorsal closure, spatiotemporal regulation of cytoskeletal tension drives precise matching of segmentation gene expression patterns across the fusing epithelial flanks [75]. And, two, during heart morphogenesis, differential expression of adhesion proteins ensure precise cell matching by modulating filopodia binding dynamics in contralateral cardioblasts [76]. Taken together, across diverse systems, the mechanical properties of cells and tissues play a vital role in ensuring robustness during morphogenesis.

## Summary and outlook

While it is readily apparent that a specific tissue forms with reproducible shapes and sizes across individuals from the same species, we are just beginning to understand how precision emerges in tissue morphology during embryo development. Given that noise is inevitable in biological systems, to understand the emergence of precision, mechanisms that buffer noise, therefore, need to be studied in detail. Studying transcription and signaling network topologies that suppress noise is important, but at the same time, the role of mechanics in ensuring increased precision should also be taken into account as illustrated through several examples mentioned in this review.

One aspect I have conveniently ignored here is the importance of scaling in achieving a precise size in a tissue relative to the overall body size, which is referred to as morphological allometry [77]. In several contexts, during the growth phase of a tissue, morphogen gradients have in fact been shown to scale with tissue size suggesting that a tissue can read its own size as it grows [78–80]. However, the details of the underlying mechanism is unknown and it is also yet to be clearly established if mechanics plays a role in regulating scaling properties during development particularly in the context of precision.

In general, to understand the role of mechanics, quantitative analysis of shape changes in a developing tissue need to be performed. This is possible only by performing long time-lapse imaging of developing tissues with techniques that lead to negligible phototoxicity such as light-sheet microscopy [81] and coupling such imaging modules with advanced image analysis algorithms. Furthermore, in addition to existing methodologies [82] for measuring mechanical properties such as ferrofluid droplets [83] and actuators [84], newer tools need to be developed that can allow probing the mechanical state of tissues directly *in vivo*. These tools need to be standardized in multiple systems and made widely available that can be utilized in a user-friendly manner, which will enable comparisons of mechanical properties across diverse tissues using the same tools. Importantly, such quantitative efforts need to be coupled with the development of mechanical models to provide a deeper understanding of mechanisms that ensure the precision of tissue shapes and sizes.

## Perspectives

- It is well known that investigating the mechanical properties of cells and tissues is vital for understanding tissue morphogenesis. It is now increasingly appreciated that tissue mechanics is not just important to mediate shape changes but also to ensure precision in the final shape and size a tissue attains.
- To emerge in a precise manner, intrinsic noise in biological processes need to be buffered. Over the next few years by studying more tissues from the perspective of precision, particularly in the context of tissue plasticity and mechanics, the similarity and diversity of noise buffering mechanisms can be better laid out.
- An in-depth understanding of how reproducible shapes emerge is likely to have a huge impact on tissue engineering as well as in generating reproducible tissues on a chip from patient-derived samples, an important bottle-neck to solve in the quest towards personalized medicine.

## Abbreviations

AP: anteroposterior
hpf: hours post fertilization
ML: mediolateral

## References

[1] Félix, M.-A. and Barkoulas, M. (2015) Pervasive robustness in biological systems. Nat. Rev. Genet. 16, 483–496 10.1038/nrg3949 26184598

[2] Wagner, A. (2005) Distributed robustness versus redundancy as causes of mutational robustness. BioEssays 27, 176–188 10.1002/bies.20170 15666345

[3] Zinani, O.Q.H., Keseroğlu, K., Ay, A. and Özbudak, E.M. (2021) Pairing of segmentation clock genes drives robust pattern formation. Nature 589, 431–436 10.1038/s41586-020-03055-0 33361814 PMC7932681

[4] Exelby, K., Herrera-Delgado, E., Garcia Perez, L., Perez-Carrasco, R., Sagner, A. and Metzis, V. et al. (2021) Precision of tissue patterning is controlled by dynamical properties of gene regulatory networks. Development 148, dev197566 10.1242/dev.197566 33547135 PMC7929933

[5] El-Samad, H. (2021) Biological feedback control—respect the loops. Cell Syst. 12, 477–487 10.1016/j.cels.2021.05.004 34139160

[6] Bhattacharya, P., Raman, K. and Tangirala, A.K. (2022) Discovering design principles for biological functionalities: perspectives from systems biology. J. Biosci. 47, 56 10.1007/s12038-022-00293-4 36222149

[7] Davidson, L.A. (2017) Mechanical design in embryos: mechanical signalling, robustness and developmental defects. Phil. Trans. R. Soc. B: Biol. Sci. 372, 20150516 10.1098/rstb.2015.0516 PMC5379024 28348252

[8] Naganathan, S.R., Popovic, M. and Oates, A.C. (2022) Left–right symmetry of zebrafish embryos requires somite surface tension. Nature 605, 516–521 10.1038/s41586-022-04646-9 35477753

[9] Eritano, A.S., Bromley, C.L., Bolea Albero, A., Schütz, L., Wen, F.-L. and Takeda, M. et al. (2020) Tissue-scale mechanical coupling reduces morphogenetic noise to ensure precision during epithelial folding. Dev. Cell 53, 212–228.e12 10.1016/j.devcel.2020.02.012 32169160

[10] Hervieux, N., Tsugawa, S., Fruleux, A., Dumond, M., Routier-Kierzkowska, A.-L. and Komatsuzaki, T. et al. (2017) Mechanical shielding of rapidly growing cells buffers growth heterogeneity and contributes to organ shape reproducibility. Curr. Biol. 27, 3468–3479.e4 10.1016/j.cub.2017.10.033 29129534

[11] Tsai, T.Y.-C., Sikora, M., Xia, P., Colak-Champollion, T., Knaut, H. and Heisenberg, C.-P. et al. (2020) An adhesion code ensures robust pattern formation during tissue morphogenesis. Science 370, 113–116 10.1101/803635 33004519 PMC7879479

[12] Martin, E., Theis, S., Gay, G., Monier, B., Rouvière, C. and Suzanne, M. (2021) Arp2/3-dependent mechanical control of morphogenetic robustness in an inherently challenging environment. Dev. Cell 56, 687–701.e7 10.1016/j.devcel.2021.01.005 33535069 PMC7955168

[13] Yevick, H.G., Miller, P.W., Dunkel, J. and Martin, A.C. (2019) Structural redundancy in supracellular actomyosin networks enables robust tissue folding. Dev. Cell 50, 586–598.e3 10.1016/j.devcel.2019.06.015 31353314 PMC7416653

[14] Oates, A.C., Morelli, L.G. and Ares, S. (2012) Patterning embryos with oscillations: structure, function and dynamics of the vertebrate segmentation clock. Development 139, 625–639 10.1242/dev.063735 22274695

[15] Oates, A.C. (2020) Waiting on the fringe: cell autonomy and signaling delays in segmentation clocks. Curr. Opin. Genet. Dev. 63, 61–70 10.1016/j.gde.2020.04.008 32505051

[16] Clark, E. (2021) Time and space in segmentation. Interface Focus 11, 20200049 10.1098/rsfs.2020.0049 34055302 PMC8086912

[17] Venzin, O.F. and Oates, A.C. (2019) What are you synching about? Emerging complexity of Notch signaling in the segmentation clock. Dev. Biol. 460, 40–54 10.1016/j.ydbio.2019.06.024 31302101

[18] Rohde, L.A., Bercowsky-Rama, A., Valentin, G., Ram Naganathan, S., Desai, R.A. and Strnad, P. et al. (2024) Cell-autonomous timing drives the vertebrate segmentation clock’s wave pattern. eLife 13, RP93764 10.7554/eLife.93764.1 PMC11643631 39671306

[19] Bajard, L., Morelli, L.G., Ares, S., Pecreaux, J., Julicher, F. and Oates, A.C. (2014) Wnt-regulated dynamics of positional information in zebrafish somitogenesis. Development 141, 1381–1391 10.1242/dev.093435 24595291 PMC3943186

[20] Moreno, T.A., Jappelli, R., Belmonte, J.C.I. and Kintner, C. (2008) Retinoic acid regulation of the Mesp–Ripply feedback loop during vertebrate segmental patterning. Dev. Biol. 315, 317–330 10.1016/j.ydbio.2007.12.038 18261720 PMC4648629

[21] Janesick, A., Tang, W., Nguyen, T.T.L. and Blumberg, B. (2017) RARβ2 is required for vertebrate somitogenesis. Development 144, 1997–2008 10.1242/dev.144345 28432217

[22] Fethullah Simsek, M., Singh Chandel, A., Saparov, D., Zinani, O.Q.H., Clason, N. and Özbudak, E.M. (2023) Periodic inhibition of Erk activity drives sequential somite segmentation. Nature 613, 153–159 10.1038/s41586-022-05527-x 36517597 PMC9846577

[23] Naganathan, S.R. and Oates, A.C. (2020) Patterning and mechanics of somite boundaries in zebrafish embryos. Semin. Cell Dev. Biol. 107, 170–178 10.1016/j.semcdb.2020.04.014 32444288

[24] Gomez, C., Özbudak, E.M., Wunderlich, J., Baumann, D., Lewis, J. and Pourquié, O. (2008) Control of segment number in vertebrate embryos. Nature 454, 335–339 10.1038/nature07020 18563087

[25] Schröter, C., Herrgen, L., Cardona, A., Brouhard, G.J., Feldman, B. and Oates, A.C. (2008) Dynamics of zebrafish somitogenesis. Dev. Dyn. 237, 545–553 10.1002/dvdy.21458 18265021

[26] Schröter, C. and Oates, A.C. (2010) Segment number and axial identity in a segmentation clock period mutant. Curr. Biol. 20, 1254–1258 10.1016/j.cub.2010.05.071 20637625

[27] Harima, Y., Takashima, Y., Ueda, Y., Ohtsuka, T. and Kageyama, R. (2013) Accelerating the tempo of the segmentation clock by reducing the number of introns in the Hes7 gene. Cell Rep. 3, 1–7 10.1016/j.celrep.2012.11.012 23219549

[28] Echeverri, K. and Oates, A.C. (2007) Coordination of symmetric cyclic gene expression during somitogenesis by Suppressor of Hairless involves regulation of retinoic acid catabolism. Dev. Biol. 301, 388–403 10.1016/j.ydbio.2006.10.003 17098223

[29] Simsek, M.F. and Özbudak, E.M. (2018) Spatial fold change of FGF signaling encodes positional information for segmental determination in zebrafish. Cell Rep. 24, 66–78.e8 10.1016/j.celrep.2018.06.023 29972792 PMC6063364

[30] Liao, B.-K., Jörg, D.J. and Oates, A.C. (2016) Faster embryonic segmentation through elevated Delta-Notch signalling. Nat. Commun. 7, 11861 10.1038/ncomms11861 27302627 PMC4912627

[31] Green, A.A., Mosaliganti, K.R., Swinburne, I.A., Obholzer, N.D. and Megason, S.G. (2017) Recovery of shape and size in a developing organ pair: shape and size recovery in developing organs. Dev. Dyn. 246, 451–465 10.1002/dvdy.24498 28295855 PMC5426968

[32] Mosaliganti, K.R., Swinburne, I.A., Chan, C.U., Obholzer, N.D., Green, A.A. and Tanksale, S. et al. (2019) Size control of the inner ear via hydraulic feedback. eLife 8, e39596 10.7554/eLife.39596 31571582 PMC6773445

[33] Allard, P. and Tabin, C.J. (2009) Achieving bilateral symmetry during vertebrate limb development. Semin. Cell Dev. Biol. 20, 479–484 10.1016/j.semcdb.2008.10.011 19027866

[34] Packard, D.S. and Jacobson, A.G. (1979) Analysis of the physical forces that influence the shape of chick somites. J. Exp. Zool. 207, 81–92 10.1002/jez.1402070109 438762

[35] Tlili, S., Yin, J., Rupprecht, J.-F., Mendieta-Serrano, M.A., Weissbart, G. and Verma, N. et al. (2019) Shaping the zebrafish myotome by intertissue friction and active stress. Proc. Natl Acad. Sci. U.S.A. 116, 25430–25439 10.1073/pnas.1900819116 31772022 PMC6925982

[36] Das, D., Chatti, V., Emonet, T. and Holley, S.A. (2017) Patterned disordered cell motion ensures vertebral column symmetry. Dev. Cell 42, 170–180.e5 10.1016/j.devcel.2017.06.020 28743003 PMC5568629

[37] Mongera, A., Rowghanian, P., Gustafson, H.J., Shelton, E., Kealhofer, D.A. and Carn, E.K. et al. (2018) A fluid-to-solid jamming transition underlies vertebrate body axis elongation. Nature 561, 401–405 10.1038/s41586-018-0479-2 30185907 PMC6148385

[38] Atia, L., Fredberg, J.J., Gov, N.S. and Pegoraro, A.F. (2021) Are cell jamming and unjamming essential in tissue development? Cells Dev. 168, 203727 10.1016/j.cdev.2021.203727 34363993 PMC8935248

[39] Kim, S., Pochitaloff, M., Stooke-Vaughan, G.A. and Campàs, O. (2021) Embryonic tissues as active foams. Nat. Phys. 17, 859–866 10.1038/s41567-021-01215-1 34367313 PMC8336761

[40] Bonakdar, N., Gerum, R., Kuhn, M., Spörrer, M., Lippert, A. and Schneider, W. et al. (2016) Mechanical plasticity of cells. Nat. Mater. 15, 1090–1094 10.1038/nmat4689 27376682

[41] Bi, D., Yang, X., Cristina Marchetti, M. and Lisa Manning, M. (2016) Motility-driven glass and jamming transitions in biological tissues. Phys. Rev. X 6, 021011 10.1103/PhysRevX.6.021011 28966874 PMC5619672

[42] Roberto, Cerbino and Véronique, Trappe (2023) Introduction to viscoelasticity and plasticity, and their relation to the underlying microscopic dynamics in soft matter systems. Physica A: Stat. Mech. Appl. 631, 128653 10.1016/j.physa.2023.128653

[43] Heisenberg, C.-P. and Bellaïche, Y. (2013) Forces in tissue morphogenesis and patterning. Cell 153, 948–962 10.1016/j.cell.2013.05.008 23706734

[44] Chugh, P. and Paluch, E.K. (2018) The actin cortex at a glance. J. Cell Sci. 131, jcs186254 10.1242/jcs.186254 30026344 PMC6080608

[45] Guirao, B. and Bellaïche, Y. (2017) Biomechanics of cell rearrangements in Drosophila. Curr. Opin. Cell Biol. 48, 113–124 10.1016/j.ceb.2017.06.004 28732313

[46] Clarke, D.N. and Martin, A.C. (2021) Actin-based force generation and cell adhesion in tissue morphogenesis. Curr. Biol. 31, R667–R680 10.1016/j.cub.2021.03.031 34033797 PMC8276969

[47] Yamada, K.M., Doyle, A.D. and Lu, J. (2022) Cell–3D matrix interactions: recent advances and opportunities. Trends Cell Biol. 32, 883–895 10.1016/j.tcb.2022.03.002 35410820 PMC9464680

[48] Anlaş, A.A. and Nelson, C.M. (2018) Tissue mechanics regulates form, function, and dysfunction. Curr. Opin. Cell Biol. 54, 98–105 10.1016/j.ceb.2018.05.012 29890398 PMC6214752

[49] Vignes, H., Vagena-Pantoula, C. and Vermot, J. (2022) Mechanical control of tissue shape: cell-extrinsic and -intrinsic mechanisms join forces to regulate morphogenesis. Semin. Cell Dev. Biol. 130, 45–55 10.1016/j.semcdb.2022.03.017 35367121

[50] Genuth, M.A. and Holley, S.A. (2020) Mechanics as a means of information propagation in development. BioEssays 42, 2000121 10.1002/bies.202000121 PMC7722802 32885468

[51] Mongera, A., Michaut, A., Guillot, C., Xiong, F. and Pourquié, O. (2019) Mechanics of anteroposterior axis formation in vertebrates. Annu. Rev. Cell Dev. Biol. 35, 259–283 10.1146/annurev-cellbio-100818-125436 31412208 PMC7394480

[52] Gross, P., Vijay Kumar, K. and Grill, S.W. (2017) How active mechanics and regulatory biochemistry combine to form patterns in development. Annu. Rev. Biophys. 46, 337–356 10.1146/annurev-biophys-070816-033602 28532214

[53] Le, H.A. and Mayor, R. (2023) Cell–matrix and cell–cell interaction mechanics in guiding migration. Biochem. Soc. Trans. 51, 1733–1745 10.1042/BST20230211 37610008 PMC10586762

[54] Martin, A.C. and Goldstein, B. (2014) Apical constriction: themes and variations on a cellular mechanism driving morphogenesis. Development 141, 1987–1998 10.1242/dev.102228 24803648 PMC4011084

[55] He, B., Doubrovinski, K., Polyakov, O. and Wieschaus, E. (2014) Apical constriction drives tissue-scale hydrodynamic flow to mediate cell elongation. Nature 508, 392–396 10.1038/nature13070 24590071 PMC4111109

[56] Morita, H., Grigolon, S., Bock, M., Gabriel Krens, S.F., Salbreux, G. and Heisenberg, C.-P. (2017) The physical basis of coordinated tissue spreading in zebrafish gastrulation. Dev. Cell 40, 354–366.e4 10.1016/j.devcel.2017.01.010 28216382 PMC5364273

[57] Petridou, N.I., Grigolon, S., Salbreux, G., Hannezo, E. and Heisenberg, C.-P. (2019) Fluidization-mediated tissue spreading by mitotic cell rounding and non-canonical Wnt signalling. Nat. Cell Biol. 21, 169–178 10.1038/s41556-018-0247-4 30559456

[58] Jain, A., Ulman, V., Mukherjee, A., Prakash, M., Cuenca, M.B. and Pimpale, L.G. et al. (2020) Regionalized tissue fluidization is required for epithelial gap closure during insect gastrulation. Nat. Commun. 11, 5604 10.1038/s41467-020-19356-x 33154375 PMC7645651

[59] Saadaoui, M., Rocancourt, D., Roussel, J., Corson, F. and Gros, J. (2020) A tensile ring drives tissue flows to shape the gastrulating amniote embryo. Science 367, 453–458 10.1126/science.aaw1965 31974255

[60] Lawton, A.K., Nandi, A., Stulberg, M.J., Dray, N., Sneddon, M.W. and Pontius, W. et al. (2013) Regulated tissue fluidity steers zebrafish body elongation. Development 140, 573–582 10.1242/dev.090381 23293289 PMC3561786

[61] Wang, X., Merkel, M., Sutter, L.B., Erdemci-Tandogan, G., Lisa Manning, M. and Kasza, K.E. (2020) Anisotropy links cell shapes to tissue flow during convergent extension. Proc. Natl Acad. Sci. U.S.A. 117, 13541–13551 10.1073/pnas.1916418117 32467168 PMC7306759

[62] Erdemci-Tandogan, G., Clark, M.J., Amack, J.D. and Lisa Manning, M. (2018) Tissue flow induces cell shape changes during organogenesis. Biophys. J. 115, 2259–2270 10.1016/j.bpj.2018.10.028 30455043 PMC6289824

[63] Founounou, N., Farhadifar, R., Collu, G.M., Weber, U., Shelley, M.J. and Mlodzik, M. (2021) Tissue fluidity mediated by adherens junction dynamics promotes planar cell polarity-driven ommatidial rotation. Nat. Commun. 12, 6974 10.1038/s41467-021-27253-0 34848713 PMC8632910

[64] Tetley, R.J., Staddon, M.F., Heller, D., Hoppe, A., Banerjee, S. and Mao, Y. (2019) Tissue fluidity promotes epithelial wound healing. Nat. Phys. 15, 1195–1203 10.1038/s41567-019-0618-1 31700525 PMC6837871

[65] Alert, R. and Trepat, X. (2020) Physical models of collective cell migration. Annu. Rev. Condens. Matter Phys. 11, 77–101 10.1146/annurev-conmatphys-031218-013516

[66] Barriga, E.H., Franze, K., Charras, G. and Mayor, R. (2018) Tissue stiffening coordinates morphogenesis by triggering collective cell migration in vivo. Nature 554, 523–527 10.1038/nature25742 29443958 PMC6013044

[67] Marchant, C.L., Malmi-Kakkada, A.N., Espina, J.A. and Barriga, E.H. (2022) Cell clusters softening triggers collective cell migration in vivo. Nat. Mater. 21, 1314–1323 10.1038/s41563-022-01323-0 35970965 PMC9622418

[68] Rost, F., Eugster, C., Schroter, C., Oates, A.C. and Brusch, L. (2014) Chevron formation of the zebrafish muscle segments. J. Exp. Biol. 217, 3870–3882 10.1242/jeb.102202 25267843 PMC4213178

[69] Yin, J., Lee, R., Ono, Y., Ingham, P.W. and Saunders, T.E. (2018) Spatiotemporal coordination of FGF and Shh signaling underlies the specification of myoblasts in the zebrafish embryo. Dev. Cell 46, 735–750.e4 10.1016/j.devcel.2018.08.024 30253169

[70] Niloy, R.A., Holcomb, M.C., Thomas, J.H. and Blawzdziewicz, J. (2023) The mechanics of cephalic furrow formation in the Drosophila embryo. Biophys. J. 122, 3843–3859 10.1016/j.bpj.2023.08.003 37571824 PMC10560681

[71] Aliee, M., Röper, J.-C., Landsberg, K.P., Pentzold, C., Widmann, T.J. and Jülicher, F. et al. (2012) Physical mechanisms shaping the Drosophila dorsoventral compartment boundary. Curr. Biol. 22, 967–976 10.1016/j.cub.2012.03.070 22560616

[72] Monier, B., Pélissier-Monier, A., Brand, A.H. and Sanson, B. (2010) An actomyosin-based barrier inhibits cell mixing at compartmental boundaries in Drosophila embryos. Nat. Cell Biol. 12, 60–65 10.1038/ncb2005 19966783 PMC4016768

[73] Fagotto, F., Rohani, N., Touret, A.-S. and Li, R. (2013) A molecular base for cell sorting at embryonic boundaries: contact inhibition of cadherin adhesion by ephrin/Eph-dependent contractility. Dev. Cell 27, 72–87 10.1016/j.devcel.2013.09.004 24094740

[74] Hong, L., Dumond, M., Tsugawa, S., Sapala, A., Routier-Kierzkowska, A.-L. and Zhou, Y. et al. (2016) Variable cell growth yields reproducible organ development through spatiotemporal averaging. Dev. Cell 38, 15–32 10.1016/j.devcel.2016.06.016 27404356

[75] Das Gupta, P.T. and Narasimha, M. (2019) Cytoskeletal tension and Bazooka tune interface geometry to ensure fusion fidelity and sheet integrity during dorsal closure. eLife 8, e41091 10.7554/eLife.41091 30995201 PMC6469929

[76] Zhang, S., Amourda, C., Garfield, D. and Saunders, T.E. (2018) Selective filopodia adhesion ensures robust cell matching in the Drosophila heart. Dev. Cell 46, 189–203.e4 10.1016/j.devcel.2018.06.015 30016621

[77] Mirth, C.K., Anthony Frankino, W. and Shingleton, A.W. (2016) Allometry and size control: what can studies of body size regulation teach us about the evolution of morphological scaling relationships? Curr. Opin. Insect. Sci. 13, 93–98 10.1016/j.cois.2016.02.010 27436558

[78] Romanova-Michaelides, M., Aguilar-Hidalgo, D., Jülicher, F. and Gonzalez-Gaitan, M. (2015) The wing and the eye: a parsimonious theory for scaling and growth control? The wing and the eye. Wiley Interdiscip. Rev. Dev. Biol. 4, 591–608 10.1002/wdev.195 26108346

[79] Čapek, D. and Müller, P. (2019) Positional information and tissue scaling during development and regeneration. Development 146, dev177709 10.1242/dev.177709 31862792

[80] Wartlick, O., Mumcu, P., Kicheva, A., Bittig, T., Seum, C. and Jülicher, F. et al. (2011) Dynamics of Dpp signaling and proliferation control. Science 331, 1154–1159 10.1126/science.1200037 21385708

[81] Wan, Y., McDole, K. and Keller, P.J. (2019) Light-sheet microscopy and its potential for understanding developmental processes. Annu. Rev. Cell Dev. Biol. 35, 655–681 10.1146/annurev-cellbio-100818-125311 31299171

[82] Campàs, O. (2016) A toolbox to explore the mechanics of living embryonic tissues. Semin. Cell Dev. Biol. 55, 119–130 10.1016/j.semcdb.2016.03.011 27061360 PMC4903887

[83] Serwane, F., Mongera, A., Rowghanian, P., Kealhofer, D.A., Lucio, A.A. and Hockenbery, Z.M. et al. (2016) In vivo quantification of spatially varying mechanical properties in developing tissues. Nat. Methods 14, 181–186 10.1038/nmeth.4101 27918540 PMC5524219

[84] Özkale, B., Parreira, R., Bekdemir, A., Pancaldi, L., Özelçi, E. and Amadio, C. et al. (2019) Modular soft robotic microdevices for dexterous biomanipulation. Lab Chip 19, 778–788 10.1039/C8LC01200H 30714604 PMC6394202

